# Airflow Obstruction in Adults with Williams Syndrome and Mice with Elastin Insufficiency

**DOI:** 10.3390/diagnostics12061438

**Published:** 2022-06-10

**Authors:** Elise K. Kronquist, Maninder Kaur, Leah M. Gober, Russell H. Knutsen, Yi-Ping Fu, Zu-Xi Yu, Danielle R. Donahue, Marcus Y. Chen, Sharon Osgood, Neelam Raja, Mark D. Levin, Amisha Barochia, Beth A. Kozel

**Affiliations:** 1 National Heart Lung and Blood Institute, National Institutes of Health, Bethesda, MD 20892, USA; elise.kronquist@nih.gov (E.K.K.); maninder.kaur@nih.gov (M.K.); leah.m.gober@gmail.com (L.M.G.); russell.knutsen@nih.gov (R.H.K.); yi-ping.fu@nih.gov (Y.-P.F.); yuz@nhlbi.nih.gov (Z.-X.Y.); chenmy@nhlbi.nih.gov (M.Y.C.); sharon.osgood@nih.gov (S.O.); neelam.raja@nih.gov (N.R.); mark.levin@nih.gov (M.D.L.); amisha.barochia@nih.gov (A.B.); 2 National Institute of Neurological Disorders and Stroke, National Institutes of Health, Bethesda, MD 20824, USA; donahued@mail.nih.gov

**Keywords:** Williams Beuren syndrome, pulmonary function tests, air trapping, obstructive pulmonary disease

## Abstract

Williams–Beuren syndrome (WS) results from the deletion of 25–27 coding genes, including elastin (*ELN*), on human chromosome 7q11.23. Elastin provides recoil to tissues; emphysema and chronic obstructive pulmonary disease have been linked to its destruction. Consequently, we hypothesized that elastin insufficiency would predispose to obstructive features. Twenty-two adults with WS (aged 18–55) and controls underwent pulmonary function testing, 6 min walk, and chest computed tomography (CT). Lung and airspace dimensions were assessed in *Eln^+/−^* and control mice via microCT and histology. The forced expiratory volume in 1 s (FEV_1_) and the ratio of FEV_1_ to forced vital capacity (FVC) were lower in adults with WS (*p* < 0.0001 and *p* < 0.05, respectively). The FEV_1_/FVC ratio was more frequently below the lower limit of normal in cases (*p* < 0.01). The ratio of residual volume to total lung capacity (RV/TLC, percent predicted) was higher in cases (*p* < 0.01), suggesting air trapping. People with WS showed reduced exercise capacity (*p* < 0.0001). In *Eln^+/−^* mice, ex vivo lung volumes were increased (*p* < 0.0001), with larger airspaces (*p* < 0.001). Together these data show that elastin insufficiency impacts lung physiology in the form of increased air trapping and obstruction, suggesting a role for lung function monitoring in adults with WS.

## 1. Introduction

Elastin is an extracellular matrix protein that provides recoil to tissues that stretch, including the lungs [1,2]. It is expressed during a restricted developmental window but makes up 20–30% of the lung’s dry weight [3]. At the same time, it turns over slowly, with a half-life of 74 years [4], suggesting the possibility that elastin insufficiency may be associated with not only developmental abnormalities but also progressive symptoms associated with elastin turnover that may be uncovered in later life.

Single nucleotide variants in the 3′ end of *ELN* (the elastin gene) cause autosomal dominant cutis laxa (MIM #123700), which is associated with severe emphysema and characteristically lax skin [5,6]. Additionally, isolated severe chronic obstructive pulmonary disease (COPD) was described in two pedigrees with the c.2318 G > A; p.G773D variant in the extreme C-terminus [7,8]. More recently, a proband and his father were identified with a variant in the start codon of *ELN* (c.2T > C; p.M1T), causing elastin haploinsufficiency and supravalvular aortic stenosis (SVAS, MIM #185500) [9]. In that pedigree, the father was diagnosed with early onset emphysema, exacerbated by smoking, and required a lung transplant at age 45 [9]. The paternal grandfather, also with emphysema and cardiac disease, was not genetically evaluated.

In addition to the single gene defects, elastin-mediated disease also occurs through a contiguous deletion on human chromosome 7q11.23 that removes 25–27 genes, including *ELN*: Williams syndrome (Williams–Beuren syndrome, WS, MIM #194050) [10]. The condition occurs in 1:7500 live births [11], with elastin-mediated disease features including SVAS and early skin wrinkling [12,13,14]. While rare cases of congenital lobar [15,16] or adult onset emphysema [17,18] in people with WS have been published, cohort data has been limited. One study presented 16 young adults with WS and an average age of 20 years [17]. In this group, forced expiratory volume in 1 s (FEV_1_, the only spirometry value reported) was normal. The authors also reported increased shortness of breath, wheezing, and coughing among the group [17]. The second WS study included 22 affected people with an average age of 18.9 years [19]. Average pulmonary function testing (PFT) measurements for this group were also normal [19]. However, two individuals had evidence of restriction while six had obstructive findings [19].

Likewise, reported lung phenotypes in elastin insufficient (*Eln^+/−^*) mice were also mild, revealing altered stress-strain relationships on mechanical testing, but statistically normal lung volumes and air space sizes [20]. More severe outcomes were noted in mutants with either a further reduction of elastin (*Eln*^−/−^; *hBAC ELN+*, possessing ~30% of the expected elastin) or in *Eln^+/−^* mice stressed by exposure to cigarette smoke [20].

All in all, previous studies have shown surprisingly little impact of elastin insufficiency on lung function. However, all humans and animals described to date have been relatively young and the impact of elastin insufficiency in older individuals is not yet known. Consequently, we performed PFTs in a new group of individuals with WS with a broader and older age distribution to address this disparity. Because human tissue is difficult to obtain (especially in the rare disease setting), we paired the human PFT analysis with imaging and histology in the *Eln^+/−^* mouse. By focusing on the *Eln*^+/−^ mutant, we were able to focus on the functional consequences of this specific gene and to assess tissue effects more directly than can be done in live patients, allowing improved mechanistic insight. The incorporation of an automated method for calculation of alveolar size provides improved precision for these studies.

## 2. Results

### 2.1. Human Subjects’ Characteristics

Table 1 reports the demographic data for the participants performing PFTs in the study. Demographic data for further tests (e.g., 6 min walk and CT) can be found in Table S1 but all show that cases and controls were well matched for age, sex, and body mass index; however, a smaller body surface area (BSA) area was noted, as expected, for those with WS. The percent of the cohort that was white was higher for those with WS, but not statistically so. No cases or controls were current or previous smokers. Median and interquartile range are shown in the table due to the non-Gaussian distribution of the data, but the mean age for comparison to previous studies is 29.0 (range 18.1–55.2) years for cases and 33.2 (range 20–54.8) years for controls.

### 2.2. Lungs of Patients with WS Show Evidence of Abnormal Lung Function with a Predominantly Obstructive Pattern

To evaluate lung mechanics in people with WS as compared to controls, we performed pulmonary function testing. Table 2 reports the numbers of patients and controls who fell outside the normal range for several PFT variables. Eight individuals with WS and one control had forced expiratory volume in 1 s/forced vital capacity (FEV1/FVC) ratio values below the lower limit of normal (LLN), with obstructive [6] or mixed [2] ventilatory defects ranging from mild to severe in cases, and an obstructive ventilatory defect in one control subject, who had a history of asthma (36.4% of cases vs. 4.5% of controls, *p* < 0.01; Chi-square analysis). Additionally, 10 WS cases and 2 control subjects had evidence of air trapping as defined by residual volume (RV)/ total lung capacity (TLC) % predicted or RV % predicted ≥120% (47.6% of cases vs. 9.1% of controls, *p* < 0.01; Chi-square analysis). One individual with WS had a mild restrictive ventilatory defect (TLC < LLN). Additionally, we identified one patient with WS who had a clinical history of asthma and a third had a chest wall abnormality (pectus excavatum) that had been surgically corrected, but although that third individual had a FVC < LLN and evidence of air trapping, TLC was normal. One control individual also had a mild restrictive lung pattern (FVC and TLC < LLN). Those with WS and appreciable PFT abnormalities ranged in age from 20.1–55.2 years, with a median of 23.9 years (mean of 29.2 years). Taken together, 16 (8 obstructive/mixed + 7 air trapping + 1 restrictive) of 22 individuals in the WS cohort (72.7%) had at least one abnormality noted on PFT as compared to 4 (1 obstructive/mixed + 2 air trapping + 1 restrictive) of 22 controls (18.2%; *p* < 0.001, Chi-square analysis).

**Figure 1:**
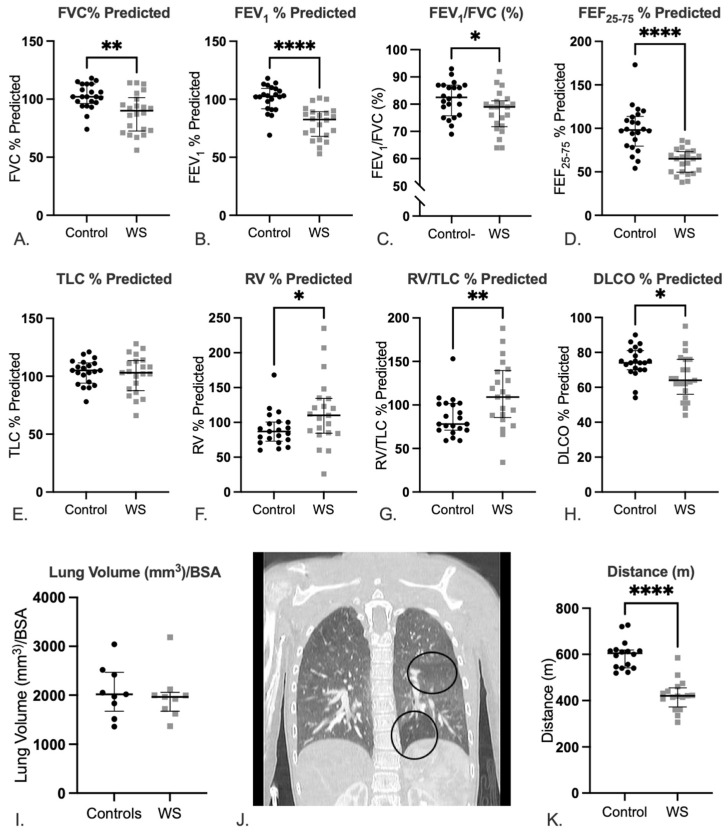
Pulmonary evaluation reveals reduced lung function and air trapping in cases compared to controls. Spirometry and lung volume measurements in WS cases and healthy controls: (**A**) FVC, (**B**) FEV_1_, (**C**) FEV_1_/FVC, (**D**) FEF_25–75_, (**E**) TLC, (**F**) RV, (**G**) RV/TLC, and (**H**) DLCO are shown (all are presented as percentage predicted values except FEV_1_/FVC [%]). All data are presented as median and interquartile range. 21/22 cases completed RV, RV/TLC, and DLCO testing. Statistics are reported as Mann–Whitney tests. *p* values reported are * *p* < 0.05, ** *p* < 0.01, and **** *p* < 0.0001. CT analyses show adults with WS have normal lung volumes: (**I**) Lung volumes assessed via CT and normalized to BSA, but a subset show evidence of air trapping: (**J**) Representative image of focal lucency in the base of the left lung and an additional heterogeneous area in the left mid-lung in a patient with WS. Statistics are reported as Mann–Whitney test. At the same time, (**K**) individuals with WS have significantly shorter 6 min walk distances compared to controls. Statistics are reported as a Mann–Whitney test. For all tests: *p* values reported are * *p* < 0.05, ** *p* < 0.01, and **** *p* < 0.0001. Abbreviations: FVC: forced vital capacity, FEV_1_: forced expiratory volume in 1 s, FEF_25–75_: Forced expiratory flow between 25 and 75%, TLC: total lung capacity, RV: residual volume, DLCO: diffusion capacity for carbon monoxide, BSA: body surface area.

Taken as a group, people with WS exhibit lower percentage predicted values for FVC (Figure 1A, control median: 113, interquartile range (IQR): 16 vs. WS median: 101.3, IQR: 24; *p* = 0.002) and FEV_1_ (Figure 1B, control median: 109.3, IQR: 16 vs. WS median: 89.25, IQR: 18.5; *p* < 0.0001) than controls, and the FEV_1_/FVC ratio is decreased in cases (Figure 1C, control median: 87.0, IQR: 10.5 vs. WS median: 81.3, IQR: 13; *p* = 0.04), supporting a signal for obstructive pattern on spirometry for people with the WS diagnosis. Forced expiratory flow between 25 and 75% (FEF_25–75_) is also decreased in cases compared to controls (Figure 1D, control median: 113.8, IQR: 75 vs. WS median: 73.3, IQR: 21; *p* < 0.0001) further supporting airflow obstruction. There is no difference in total lung capacity (TLC, % predicted) (Figure 1E, control median: 111.3, IQR: 16.5 vs. WS median: 113.5, IQR: 25; *p* = NS), but residual volume (RV, % predicted) is significantly greater in those with WS as compared to controls (Figure 1F, control median: 100.3, IQR: 82.5 vs. WS median: 134.5, IQR: 125; *p* = 0.03). The RV/TLC ratio (% predicted) is significantly greater in WS cases (Figure 1G, control median: 101.3, IQR: 75 vs. WS median: 139.5, IQR: 79; *p* = 0.003), suggestive of greater air trapping. Individuals with WS showed a reduction in the diffusion capacity for carbon monoxide (DLCO) (Figure 1H, control median: 81, IQR: 16 vs. WS median: 76, IQR: 31; *p* = 0.01), indicating mildly decreased gas exchange.

A subset of WS cases (*n* = 19) also underwent pulmonary imaging using computed tomography (CT). All patient CTs were assessed for lung abnormalities, but only 9 cases were assessed for lung volumes to match the 9 controls who had undergone CT. As in the PFT study, normalized lung volumes were not different between groups (Figure 1I, control median: 2019, IQR: 796 vs. WS median: 1963, IQR: 387; *p* = NS). In most cases, a review of the CT images revealed no visually obvious air trapping. However, 3 individuals exhibited heterogeneity in the lung parenchyma, with focal radiolucent areas suggestive of air trapping in the bases of the lungs (Figure 1J), and one had small subpleural blebs bilaterally.

### 2.3. Patients with WS Show Decreased Exercise Tolerance

Participants with WS walked significantly shorter distances than controls in the 6 min walk test (Figure 1K, control median: 605, IQR: 77.3 ft vs. WS median: 420, IQR: 82.5 ft; *p* < 0.0001), revealing impaired exercise capacity; however, there was no difference in oxygen saturation or significant desaturation during the test (data not shown).

### 2.4. Lungs from Elastin-Insufficient Mice Exhibit Biomechanical Differences

**Figure 2:**
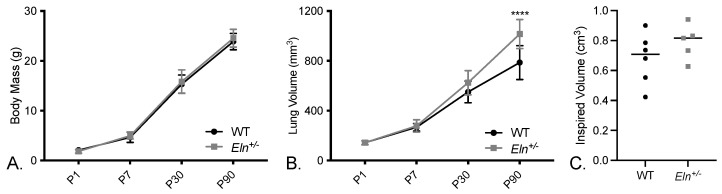
*Eln^+/−^* mice lungs are larger when assessed ex vivo, but not in vivo. (**A**) Body mass with age. (**B**) Ex vivo lung volumes. (**C**) Inspired lung volume as measured by in vivo CT at ~90 days of age. (**A**,**B**) were analyzed using two-way ANOVA with multiple corrections in (**B**) done by Šídák’s multiple comparison test. (**A**) 2-way ANOVA *p* < 0.0001 for age and *p* = NS for Eln genotype. (**B**) 2-way ANOVA, age effect *p* < 0.0001, Eln genotype effect *p* < 0.0001. **** *p* < 0.0001. (**C**) was assessed by a Mann–Whitney test (WT 0.6797 ± 0.1703 vs. *Eln^+/−^* 0.7896 ± 0.1173, *p* = NS).

To address the potential lung parenchymal differences noted in the patients with WS and elastin insufficiency, we evaluated lung volumes in *Eln^+/−^* and wild type (WT) mice. In the ex vivo microCT (uCT), we evaluated lung size over time in concurrence with body mass. Between P1 and ~P90, both groups of mice (WT and *Eln^+/−^*) showed the expected increase in body size (Figure 2A, 2-way ANOVA *p* < 0.0001 for age and *p* = NS for Eln genotype). Ex vivo lung volumes also increase with age, but more so in the *Eln^+/−^* mice (Figure 2B, 2-way ANOVA, age effect *p* < 0.0001, Eln genotype effect *p* < 0.0001) such that by 3 months of age (P90), pressure fixation in the open chest cavity reveals statistically larger lungs (*p* < 0.0001).

Alternatively, using an in vivo uCT method, similar to the human study, there was no detectable difference in inspired lung volumes between the *Eln^+/−^* and *Eln^+/+^* at 3 months of age (Figure 2C, 0.6797 ± 0.1703 vs. 0.7896 ± 0.1173, *p* = NS), suggesting that the function of the bellows (the ribcage and diaphragm) may at least partially compensate for differences in parenchymal lung biomechanics.

**Figure 3:**
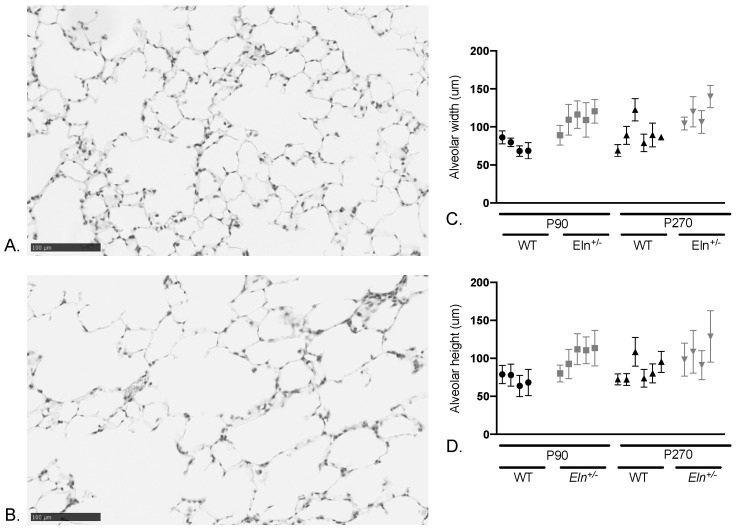
Histological analysis reveals larger airspaces in *Eln^+/−^* mouse lungs as compared to WT. Representative hematoxylin and eosin-stained image of (**A**) WT and (**B**) *Eln^+/−^* right lower lung. Graphical representation of *Eln^+/−^* and WT airspace width (**C**) and height (**D**) measured by mean linear intercept (mean +/− SD) and plotted for each animal. *Eln^+/−^* animals at age P90 and P270 are indicated in gray and WT in black.

Table 3 shows linear mixed effects model analysis of peripheral airspace size of ~90 and ~270 day WT and *Eln^+/−^* mice (Figure 3A,B) confirms larger airspaces in the ex vivo *Eln^+/−^* lungs compared to WT (Figure 3C,D; *p* < 0.0001).

## 3. Discussion

Elastin aids in lung recoil, influencing the total amount of air that remains in the lung after expiration. Given the reduction in lung elastin for people with WS and elastin haploinsufficiency, there is an expectation that the condition could produce clinically significant pulmonary disease. Surprisingly, previous descriptions revealed only mild pulmonary impact [17,19]. Explanations for this finding include the possibility that ~50% of the normal elastin content remains adequate for normal lung function—or at least that at this level, the bellows and chest wall are able to compensate. As such, one might hypothesize that additional turnover of elastin (the half-life of elastin in the lungs is ~74 years [4]), which further reduces the already low levels of elastin to a critical threshold, or environmental exacerbators such as smoking that destroy elastin are required to produce a clinically identifiable phenotype. To date, the studies examining pulmonary function in WS were performed in relatively young cohorts, potentially limiting their ability to fully exhibit the impact of chronic elastin haploinsufficiency in the lung. Taking this into account, our study is the first to examine pulmonary function paired with CT chest analysis in a non-pediatric cohort, with an average age in the late 20s.

As a cohort, the group with WS had decreased FEV_1_/FVC compared to controls, and 8 WS cases out of 22 had an FEV_1_/FVC ratio below the lower limit of normal (LLN), indicating a higher incidence of obstructive airways disease. Additionally, 10 individuals with WS had evidence of air trapping, perhaps a more subtle indicator of obstruction in the smaller airways. These findings are evidence of a signal for airflow obstruction in patients with WS as early as a person’s mid-20s. Thus, our results are a departure from Wan et al., where pulmonary function findings were similar between case and control cohorts [17], and show more impairment than demonstrated by Pangallo et al. whose FEV_1_/FVC average was higher (82% vs. our 77%) [19]. While we are not fully able to study age as a variable due to the relative paucity of studied individuals greater than 40 years, given that the average patient age in our cohort is older than the two previous studies, it may be that age is a factor in the decreased lung function seen in our patients. Those with WS and appreciable lung function abnormalities had a median age of 23.9 (mean 29.2) years but covered a broad age range (20.1–55.2 years).

Analysis of lung volumes from chest CT reflected the PFT findings; normalized lung volumes were not greater in the WS cohort, but a subset displayed focal areas of hyperlucency or mosaicism in the lungs, consistent with a diagnosis of air trapping. However, this finding may have been underrecognized in WS patients due to limitations in CT type and quality. Because CTs were originally performed as part of a vascular investigation, they are not optimized for pulmonary analysis; specifically, they did not include end-expiratory imaging which is more sensitive for air trapping. Additionally, not all patients were able to satisfactorily complete scanning. Thus, it is possible that we are missing evidence of air trapping on some CTs as a result.

Participants with WS did not report baseline difficulty breathing; however, their 6 min walk tests revealed significant reduction in exercise capacity. While many factors determine exercise capacity, the pulmonary function abnormalities described here may contribute to this outcome. Given that oxygen saturation was not decreased in individuals with WS, it is also possible that neuromuscular or cardiovascular differences may contribute to impaired exercise tolerance.

Parallel imaging and histological studies in *Eln^+/−^* mice confirm normal in vivo lung size, but increased ex vivo volumes for lungs filled under standard pressures. Taken together, these findings suggest a difference in parenchymal lung mechanics due to elastin insufficiency that may be overcome by chest wall dynamics. These results parallel the findings by Shifren et al. that show increased lung compliance in the *Eln^+/−^* animals [20]. Biochemically, they confirmed elastin crosslinking was reduced ~45% in the *Eln^+/−^* [20]. Their histological analysis, however, demonstrated no difference in peripheral airspace size between genotypes, a finding they attributed to maintenance of collagen content in the lungs [20], while our assessment revealed significantly larger airspaces in *Eln^+/−^* mice compared to WT. This may be due to different genetic backgrounds (our *Eln^+/−^* mice were backcrossed to C57Bl/6 to reduce the contaminating 129x1/Sv material in the parental line [21]), increased power, or differences in technique (CT quantification for lung size and automated image-based modalities for the measurement of peripheral airspace size [22]). While a trend for larger airspaces was seen in the mouse study, it did not reach significance with the number of mice used.

This study is limited in a few important ways. First, it is possible that pulmonary abnormalities only manifest at an older age in WS, and although this cohort is older than previous reported groups and includes patients up to their mid-50s, there are still only a limited number of participants over age 30. Further, our research study requires participants to travel to the NIH for testing. As such, we are missing the oldest patients and potentially those with the most severe pulmonary disease (e.g., those who utilize supplemental oxygen and/or are unable to travel due to disease severity), giving us a selection bias towards relatively healthy individuals. Likewise, while 9 month old mice may be “older” than the 3 month old mice often studied, they may not be old enough to experience significant turnover of elastin, especially given the estimated half-life of elastin in rodents is 27–40 years [23]. Additionally, environmental factors such as smoking or pollution that may synergize with elastin insufficiency to accelerate pulmonary disease [9,20], were not evaluated in the current study.

## 4. Materials and Methods

Additional methodological details can be found in the Supplemental methods.

### 4.1. Human Subjects’ Protections and Enrollment

Oversight was provided by the Institutional Review Board of the National Institutes of Health (NIH). People with WS and controls consented to the Impact of Elastin Mediated Vascular Stiffness on End Organs study at the NIH Clinical Center (clinical trial number: NCT02840448, last continuing review approved 15 March 2022). Additional control data were obtained under a waiver of consent. See Figure S1 for enrollment and inclusion/exclusion details. Healthy controls were matched to the WS cohort in aggregate by age and sex.

### 4.2. Pulmonary Function Testing

Participants underwent pulmonary function testing (PFT) including spirometry, lung volumes, diffusion capacity, and 6 min walk tests. Tests were performed according to American Thoracic Society guidelines [24,25,26].

### 4.3. CT Analysis of Lung Volume and Air trapping

Participants underwent chest computed tomography (CT) scans (Canon Medical, Otawara, Japan). Vitrea Advanced Visualization 7.11.5.29 (Vital Images Inc., Minnetonka, MN, USA) was used to analyze data. Parenchymal lung volume was assessed during breath hold. Lung volumes are normalized to body surface area (BSA). Acceptable CT scans were examined for other pulmonary abnormalities.

### 4.4. Animal Studies

All procedures were approved by the National Heart Lung and Blood Institute Institutional Animal Care and Use Committee. Institutional guidelines for experimentation and welfare were followed. Postnatal day (P) 1, 7, 30, ~90, and ~270 *Eln^+/−^* and *Eln^+/+^* mice in a backcrossed C57Bl/6 background were used [21,27,28].

### 4.5. Ex Vivo Processing and microCT Imaging and Analysis

Post-natal day (P) 1, 7, 30, and ~90-day old mouse lungs were pressure-fixed to 20 cm H_2_O in the open chest cavity according to previously published methods and imaged using the Quantum GX microCT (μCT) scanner (PerkinElmer, Waltham, MA, USA) [29]. Lung volumes were calculated with Amira 6.7.0 software (FEI, Hillsboro, OR, USA) following segmentation.

### 4.6. In Vivo Murine Pulmonary microCT Imaging and Analysis

Anesthesia was induced with isoflurane and the mouse was transferred to the Quantum GX uCT chamber for scanning. Cardiac and respiratory rhythms were simultaneously captured [30,31]. Horos (Horosproject.org, Annapolis, MD, USA) software was used to segment and isolate lung parenchyma for volume measurements.

### 4.7. Histological Analysis of Lung Airspaces

Lungs from P ~90 and ~270 mice were inflated and fixed as above then dehydrated in ethanol and embedded in paraffin. 5 mm sections were stained with hematoxylin and eosin. Peripheral airspace size from the right inferior lobe was quantified using ImageJ (NIH, Bethesda, MD, USA) [22]. Briefly, a series of horizontal and vertical lines was applied to converted images and the length of each was measured using a plugin [22]. Mean linear intercept (MLI) measures were obtained, and mean height and width were calculated.

### 4.8. Statistics

Analysis was performed using GraphPad Prism software, version 9.0 (GraphPad Software, San Diego, CA, USA). For human data, unpaired Mann–Whitney tests were conducted, reported as median and interquartile range (IQR). Chi-squared analysis was used where indicated. For animal data, Kruskal–Wallis tests were conducted, with multiple comparisons as reported in the figure legends. Linear mixed effects models adjusted for age were used to examine the association between genotypes and the MLI measures. Data are reported as mean +/− standard deviation with 5% significance cutoff.

## 5. Conclusions

Overall, our findings suggest that adults with WS do exhibit more impaired lung function than controls; however, their pulmonary disease is generally mild and may be missed on standard clinical evaluation unless pulmonary function testing is performed. This is the first study to focus only on adults with WS, showing a higher incidence of obstructive lung disease on pulmonary function testing as well as possible air trapping on CT of the chest. Because elastin continues to be slowly turned over and not replaced, there is a possibility of progressive air trapping and obstruction. As such, it may be important to serially monitor pulmonary function in adults to evaluate for progressive decline, especially in those who exhibit abnormalities at their initial evaluation. Smoking, of course, should be discouraged in all individuals, but avoidance may be even more important in people with elastin insufficiency—both those with Williams syndrome and those with familial ELN-related SVAS. More data are needed to determine how function changes with more advanced age, and assessment of pulmonary function may reveal further impacts of elastin insufficiency with an even older population.

## Figures and Tables

**Table 1:** Demographic data for study participants who underwent Pulmonary Function Testing (PFT). * All patients completed spirometry measures, and 21/22 cases completed lung capacity and DLCO measurements. Non-white cases identified as multiracial or “unknown” on the study questionnaire, while 9.1% of controls were Black/African American, 4.5% were Asian, and 18.2% identified as “other” or “unknown.” Non-parametric Mann–Whitney tests were performed for age, body surface area, and body mass index. Chi square tests were performed for variables including race/ethnicity and sex. Abbreviations: IQR: interquartile range, DLCO: diffusion capacity for carbon monoxide.

Pulmonary Function Testing Participants	Cases with WS	Controls	Significance
Number *	22	22	
**Age (median, IQR)**	25.0, IQR: 24.3	28.0, IQR: 14.2	*p* = 0.2
**Race/Ethnicity (% white)**	90.9	68.2	*p* = 0.06
**Sex (% male)**	36.4	36.4	*p* > 0.999
**Body surface area (median, IQR)**	1.63, IQR: 0.5	1.84, IQR: 0.2	*p* = 0.01
**Body mass index (median, IQR)**	24.1, IQR: 10.4	25.7, IQR: 5.5	*p* = 0.5
**Asthma**	1	1	*p* > 0.999

**Table 2:** Outcomes of pulmonary function testing reveal increased incidence of lung abnormalities in adults with WS compared to controls. FEV_1_/FVC < LLN occurs more frequently in cases compared to controls and is indicative of obstructive lung disease in patients with that finding. RV/TLC > 120% is more common in adults with WS compared to controls and suggests that air trapping is more common in cases. Overall, patients with WS had more findings of lung abnormalities than control counterparts. X^2^ tests were performed for all variables indicated. Abbreviations: FEV_1_: forced expiratory volume in 1 s, LLN: lower limit of normal, FVC: forced vital capacity, TLC: total lung capacity, RV/TLC: residual volume/total lung capacity, RV: residual volume.

PFT Parameter	WS	Controls	X^2^
	Number (%)		
FEV_1_ < LLN	10 (45.5)	1 (4.5)	*p* = 0.002
FVC < LLN	8 (36.4)	1 (4.5)	*p* = 0.009
FEV_1_/FVC < LLN	8 (36.4)	1 (4.5)	*p* = 0.009
TLC < LLN	1 (4.7)	1 (4.5)	*p* > 0.973
RV/TLC or RV (% predicted) > 120	10 (47.6)	2 (9.1)	*p* = 0.005
At least one of the above	16 (72.7)	4 (18.2)	*p* = 0.0003

**Table 3:** Linear mixed effects model results for horizontal intercepts. *Eln^+/−^* mouse lungs have larger airspaces, however, there is no statistically significant difference in airspace size with age. See Table S2 in the online data supplement for vertical intercept results. Abbreviations: 95% CI-L: 95% confidence interval lower limit, 95%% CI-U: confidence interval upper limit.

Variables		N	%	*p*-Value	Least Square Means	Standard Error	95% CI-L	95% CI-U
Age	Old	10	52.63	0.1107	103.73	4.5765	94.0276	113.43
	Young	9	47.37		92.485	4.787	82.337	102.63
Genotype	WT	10	52.63	2.84 × 10^−4^	82.7259	4.5765	73.0242	92.4276
	*Eln* ^+/−^	9	47.37		113.49	4.787	103.34	123.64

## Data Availability

The data presented in this study are available on request from the corresponding author.

## Supplementary Materials

The following supporting information can be downloaded at: https://www.mdpi.com/article/10.3390/diagnostics12061438/s1, Table S1: Demographic data for 6 min walk and CT subtests; Table S2: Linear mixed effects model results for vertical intercepts. Supplemental Methods; and Figure S1: Inclusion and exclusion criteria for the study.

## References

[1] Duque Lasio M.L., Kozel B.A. (2018). Elastin-driven genetic diseases. Matrix Biol..

[2] Kozel B.A., Mecham R.P. (2019). Elastic fiber ultrastructure and assembly. Matrix Biol..

[3] Mecham R.P. (2018). Elastin in lung development and disease pathogenesis. Matrix Biol..

[4] Shapiro S.D., Endicott S.K., Province M.A., Pierce J.A., Campbell E.J. (1991). Marked longevity of human lung parenchymal elastic fibers deduced from prevalence of D-aspartate and nuclear weapons-related radiocarbon. J. Clin. Investig..

[5] Rodriguez-Revenga L., Badenas C., Carrio A., Mila M. (2005). Elastin mutation screening in a group of patients affected by vascular abnormalities. Pediatr. Cardiol..

[6] Urban Z., Gao J., Pope F.M., Davis E.C. (2005). Autosomal dominant cutis laxa with severe lung disease: Synthesis and matrix deposition of mutant tropoelastin. J. Investig. Dermatol..

[7] Cho M.H., Ciulla D.M., Klanderman B.J., Hersh C.P., Litonjua A.A., Sparrow D., Raby B.A., Silverman E.K. (2009). Analysis of exonic elastin variants in severe, early-onset chronic obstructive pulmonary disease. Am. J. Respir. Cell Mol. Biol..

[8] Kelleher C.M., Silverman E.K., Broekelmann T., Litonjua A.A., Hernandez M., Sylvia J.S., Stoler J., Reilly J.J., Chapman H.A., Speizer F.E. (2005). A functional mutation in the terminal exon of elastin in severe, early-onset chronic obstructive pulmonary disease. Am. J. Respir. Cell Mol. Biol..

[9] Louw J.J., Verleden G., Gewillig M., Devriendt K. (2012). Haploinsufficiency of elastin gene may lead to familial cardiopathy and pulmonary emphysema. Am. J. Med. Genet. A.

[10] Kozel B.A., Barak B., Kim C.A., Mervis C.B., Osborne L.R., Porter M., Pober B.R. (2021). Williams syndrome. Nat. Rev. Dis. Primers.

[11] Stromme P., Bjornstad P.G., Ramstad K. (2002). Prevalence estimation of Williams syndrome. J. Child. Neurol..

[12] Beuren A.J., Apitz J., Harmjanz D. (1962). Supravalvular aortic stenosis in association with mental retardation and a certain facial appearance. Circulation.

[13] Collins R.T. (2013). Cardiovascular disease in Williams syndrome. Circulation.

[14] Kozel B.A., Bayliss S.J., Berk D.R., Waxler J.L., Knutsen R.H., Danback J.R., Pober B.R. (2014). Skin findings in Williams syndrome. Am. J. Med. Genet. Part A.

[15] Walsh T.A., Gopagondanahalli K.R., Malhotra A. (2017). Williams-Beuren Syndrome and Congenital Lobar Emphysema: Uncommon Association with Common Pathology?. Case Rep. Pediatr..

[16] Wong W., Fiorino E. (2012). A Novel Case Report of Congenital Lobar Emphysema in a Patient with Williams Syndrome. Chest.

[17] Wan E.S., Pober B.R., Washko G.R., Raby B.A., Silverman E.K. (2010). Pulmonary function and emphysema in Williams-Beuren syndrome. Am. J. Med. Genet. Part A.

[18] Wojcik M.H., Carmichael N., Bieber F.R., Wiener D.C., Madan R., Pober B.R., Raby B.A. (2017). A new diagnosis of Williams-Beuren syndrome in a 49-year-old man with severe bullous emphysema. Am. J. Med. Genet. Part A.

[19] Pangallo E., Cianci P., Favuzza F., Milani D., Vimercati C., Moretti A., Picchi R., De Paoli A., Agosti M., Selicorni A. (2020). Pulmonary function in Williams-Beuren syndrome: Spirometric data of 22 Italian patients. Am. J. Med. Genet. Part A.

[20] Shifren A., Durmowicz A.G., Knutsen R.H., Hirano E., Mecham R.P. (2007). Elastin protein levels are a vital modifier affecting normal lung development and susceptibility to emphysema. Am. J. Physiol. Lung Cell Mol. Physiol..

[21] Kozel B.A., Knutsen R.H., Ye L., Ciliberto C.H., Broekelmann T.J., Mecham R.P. (2011). Genetic modifiers of cardiovascular phenotype caused by elastin haploinsufficiency act by extrinsic noncomplementation. J. Biol. Chem..

[22] Crowley G., Kwon S., Caraher E.J., Haider S.H., Lam R., Batra P., Melles D., Liu M., Nolan A. (2019). Quantitative lung morphology: Semi-automated measurement of mean linear intercept. BMC Pulm Med..

[23] Sherratt M.J. (2009). Tissue elasticity and the ageing elastic fibre. Age.

[24] Graham B.L., Steenbruggen I., Miller M.R., Barjaktarevic I.Z., Cooper B.G., Hall G.L., Hallstrand T.S., Kaminsky D.A., McCarthy K., McCormack M.C. (2019). Standardization of Spirometry 2019 Update. An Official American Thoracic Society and European Respiratory Society Technical Statement. Am. J. Respir. Crit. Care Med..

[25] Laboratories ATSCoPSfCPF (2002). ATS statement: Guidelines for the six-minute walk test. Am. J. Respir. Crit. Care Med..

[26] Miller M.R., Hankinson J., Brusasco V., Burgos F., Casaburi R., Coates A., Crapo R., Enright P., Van Der Grinten C.P.M., Gustafsson P. (2005). Standardisation of spirometry. Eur. Respir. J..

[27] Li D.Y., Faury G., Taylor D.G., Davis E.C., Boyle W.A., Mecham R.P., Stenzel P., Boak B., Keating M.T. (1998). Novel arterial pathology in mice and humans hemizygous for elastin. J. Clin. Investig..

[28] Li D.Y., Brooke B., Davis E.C., Mecham R.P., Sorensen L.K., Boak B.B., Eichwald E., Keating M. (1998). T Elastin is an essential determinant of arterial morphogenesis. Nature.

[29] Knutsen R.H., Gober L.M., Sukinik J.R., Donahue D.R., Kronquist E.K., Levin M.D., McLean S.E., Kozel B.A. (2020). Vascular Casting of Adult and Early Postnatal Mouse Lungs for Micro-CT Imaging. J. Vis. Exp..

[30] Dinkel J., Bartling S.H., Kuntz J., Grasruck M., Kopp-Schneider A., Iwasaki M., Dimmeler S., Gupta R., Semmler W., Kauczor H.U. (2008). Intrinsic gating for small-animal computed tomography: A robust ECG-less paradigm for deriving cardiac phase information and functional imaging. Circ. Cardiovasc. Imaging.

[31] Kojonazarov B., Belenkov A., Shinomiya S., Wilchelm J., Kampschulte M., Mizuno S., Ghofrani H.A., Grimminger F., Weissmann N., Seeger W. (2018). Evaluating Systolic and Diastolic Cardiac Function in Rodents Using Microscopic Computed Tomography. Circ. Cardiovasc. Imaging.

