# Supplementary material for: Airflow Obstruction in Adults with Williams Syndrome and Mice with Elastin Insufficiency
Source: Diagnostics (Basel). 2022 Jun 10;12(6):1438. doi: 10.3390/diagnostics12061438 (PMC9221558; doi:10.3390/diagnostics12061438)
Supplement: Supplementary file 1 [file diagnostics-12-01438-s001.zip › diagnostics-1706220-supplementary.pdf]

Supplemental Methods

Human subjects' protections and enrollment

Oversight for the clinical study was provided by the Institutional Review Board of the National Institutes of Health. People with Williams syndrome (WS) and controls were enrolled in the Impact of Elastin Mediated Vascular Stiffness on End Organs study at the NIH Clinical Center (clinical trial number: NCT02840448). Between July 21, 2016 and January 14, 2021, consent was obtained from the individual or a caregiver, as appropriate, to allow for participation. During this period, 72 people with WS or SVAS (supravalvular aortic stenosis) and 41 controls consented to the study. All individuals with WS had clinical or research testing confirming the diagnosis, with a minimum of FISH positivity for elastin. Individuals with isolated ELN-associated familial SVAS and those with known atypical deletions also recruited under this protocol were not analyzed for this study (n=10). In this study, all tests were optional. Consequently, not all tests were performed by all individuals. To be included in this analysis, participants had to have completed the test reported (usually on the first study visit but occasionally on a follow-up visit), with the study meeting standard clinical quality metrics (see Figure S1 for full cohort details). PFTs could not be reliably completed by the majority of children with WS and as such, children (age < 18y) were not included in the analysis.

Controls were then matched to the remaining WS cohort in aggregate by age and sex. Additional control data were obtained through the Biomedical Translational Research Information System (BTRIS) at the NIH Clinical Center under a waiver of consent. Demographic and diagnosis data from 257 individuals were reviewed to identify BTRIS controls that were well matched with the WS cohort and were free from common or rare comorbidities (including but not limited to: bronchiectasis, sickle cell disease, lung mass or tumor, Hyper IgE syndrome, etc.) that might influence the tests being studied.

Pulmonary function testing

Participants underwent pulmonary function testing (PFT) including spirometry, lung volumes and diffusion capacity. Lung function was assessed at the NIH Clinical Center Pulmonary Function Testing laboratory following the American Thoracic Society guidelines (24, 26). Test parameters analyzed and reported include FEV_1, total lung capacity (TLC), residual volume (RV), forced vital capacity (FVC), and forced expiratory flow 25-75% (FEF_{25-75}), and diffusion capacity of the lung for carbon monoxide (DLCO) (all reported as percent predicted values of the population average (populations take into account age, sex, body size and race/ethnicity). The FEV_1 to forced vital capacity (FEV_1/FVC) and percent predicted residual volume per total lung capacity (RV/TLC) ratios are also reported.

The 6-minute walk test was performed according to ATS criteria (25).

CT analysis of lung volume and air-trapping

Cases and controls underwent neck to pelvis computed tomography (CT) scan (Canon Medical, Otawara, Japan) with iopamidol-370 (Isovue-370) (Bracco Diagnostics, Monroe Township, NJ vascular contrast Image thickness was 2 mm with 1 mm increments.

Vitrea Advanced Visualization 7.11.5.29 (Vital Images Inc., Minnetonka, MN) was used to post-process and analyze compiled CT data. For calculation of lung volume during breath holding, pulmonary analysis mode was used. Proximal bronchi were removed prior to calculation of the lung parenchymal volume. Due to the smaller body size for people with WS, lung volumes are presented as normalized to patient body surface area (BSA).

Patients with acceptable CT scans were also examined for areas of mosaic attenuation consistent with air trapping or other pulmonary abnormalities.

Animal studies

All procedures described here were approved by the Institutional Animal Care and Use Committee (IACUC) of the National Heart Lung and Blood Institute (NHLBI). Institutional guidelines for animal experimentation and welfare were followed. Postnatal day 1, 7, 30, ~90, and ~270 $Eln^{+/-}$ and $Eln^{+/+}$ mice were used (28). This mouse was originally created in a 129x1/Sv; C57Bl/6 background but was crossed to C57Bl/6 prior to these studies to remove 129x1/Sv genetic material that may influence phenotype (21, 27).

Ex vivo processing and microCT imaging and analysis of young and adult mice

As part of a separate study of the lung vasculature, Microfil^{TM} (Flow Tech Inc., Carver, MA) injections were performed as previously described (29). Postnatal day (P) 1, 7, 30, and ~90 day old mice were used for these experiments. Briefly, the anterior chest wall was removed, the trachea cannulated, and the lungs inflated to 20 cm H_2O with 10% buffered formalin. The thorax (inflated lungs, heart, and chest wall) was then placed in 10% formalin at 4° C for 24 hours. Subsequently, the lungs were separated from the thoracic cavity and imaged using the Quantum GX microCT (uCT) scanner (PerkinElmer, Waltham, MA).

Both genotypes in each group were post-processed at the same resolution (25um for P1, 30um for P7, 35um for P30, and 40um for P90). Lung volumes were assessed using Amira 6.7.0 software (FEI, Hillsboro, OR). DICOM files were uploaded into the software and opened with the “Segmentation Editor” tool. The “Threshold” tool was used to automatically isolate the lung, with additional tools used to refine the selection and ensure inclusion of the full lung parenchyma prior to volume measurement.

In vivo murine pulmonary microCT imaging and analysis

Anesthesia was briefly induced using 4% isoflurane. Each mouse was then transferred to a heated platform, ophthalmic ointment was applied, and a level plane of anesthesia was established at 2%

isoflurane. For a separate vascular study, intravenous Viscover™ ExiTron™ nano 12000 contrast agent (Miltenyi Biotec, Bergisch-Gladbach, Germany) was administered via the tail vein, but the results are not reported here. The platform with the secured mouse was transferred within the Quantum GX microCT (uCT) chamber (PerkinElmer, Waltham, MA) and a four-minute scan was obtained at 90kV and 88uA using a 0.06mm Cu and 0.5 mm Al filter. Both cardiac and respiratory rhythms are simultaneously captured to allow for cardiac or respiratory gating at the time of analysis (30, 31).

To assess lung volumes, DICOM images of respiratory-gated inspiratory scans were uploaded to Horos (Horosproject.org, Annapolis, MD) and segmented to isolate the lung parenchyma from the surrounding tissue. Using the "2D/3D segmentation tool", a threshold was applied to automatically isolate the lung tissue by the range of pixels with the same intensity. Major airways were removed to match the human lung assessment using the eraser tool. A 3D volume rendering of the remaining lung was created and the volume measured.

Histological analysis of lung airspaces

Lungs from male and female postnatal day ~90 and ~270 mice were inflated and fixed overnight as above prior to dehydration in ethanol. Lungs were then embedded in paraffin and sectioned horizontally from the ventral side to a depth of approximately 700-1000um or until a maximal area including all five lobes and the primary bronchi were observed. 5um sections were acquired and stained with hematoxylin and eosin stains. The same slide for each sample was assessed for airspace area using ImageJ software (NIH Bethesda, MD). Briefly, sections were imaged using the Hamamatsu NanoZoomer 2.0 RS digital slide scanner (Hamamatsu, Japan). Slide images were then captured at 20X using the Hamamatsu NDP.view2 viewing software (Hamamatsu, Japan). Four non-overlapping images were collected for each animal in the lower quadrant of the right inferior lobe, being sure to stay within the borders of the lung and to exclude large airways/vessels. Average airspace size was quantified by

intercept (chord) measurement as previously reported (22). Briefly, images were converted from RGB to 8-bit and a Huang threshold was applied using a preset script created by Crowley *et al.* (22). A series of horizontal and vertical lines was applied to the image and the length of each intercept between the septal tissues was measured using a second ImageJ plugin (22). Mean linear intercept (MLI) measures were obtained across 11 vertical axes and 7 horizontal axes for each slide; mean height and mean width were calculated for each axis respectively.

<u>Statistics</u>

Analysis of data was performed using GraphPad Prism software, version 8.0 (GraphPad Software, San Diego, CA). For human data, nonparametric, unpaired Mann-Whitney tests were conducted for comparisons between cases and controls. Data are reported as median and interquartile range (IQR). Chi-squared analysis was used to analyze demographic data where indicated. For animal data, nonparametric Kruskal-Wallis tests were conducted for non-normally distributed data with multiple comparisons as reported in the figure legends. Linear mixed effects models were used to examine the association between the mouse genotypes and the repeated MLI measures of mean height and mean width, with adjustment for age of the study animal. Mouse data are reported as mean +/- standard deviation. The 5% significance level was applied in all analyses.

Tables

6 Minute Walk Testing	Cases	Controls	Significance
N	16	16	
Age (median, IQR)	27.2, IQR: 19	33.2 IQR:25.6	p=0.2
Race/Ethnicity (% white)	93.8	68.8	p=0.07
Sex (% male)	62.5	62.5	p>0.999
BSA (median, IQR)	1.65*, IQR: 0.4	1.86, IQR: 0.2	p=0.04
BMI (median, IQR)	25.5, IQR: 8.3	25.7, IQR:4.9	p=0.9
**CT**	**Cases**	**Controls**	**Significance**
N	9	9	
Age (median, IQR)	33.1, IQR: 16.2	47.8, IQR: 18.7	p=0.09
Race/Ethnicity (% white)	77.8	55.6	p=0.3
Sex (% male)	22.2	22.2	p=0.999
BSA (median, IQR)	1.64*, IQR: 0.5	1.8, IQR: 0.2	p=0.3
BMI (median, IQR)	26.0, IQR: 11.1	26.6, IQR: 6.0	p>0.999

Supplemental Table S1. Demographic data for 6-minute walk and CT subtests. Subjects in subtests were well-matched for age, sex, and BMI. BSA was smaller in cases performing 6 minute walk testing, but not CT. One patient was included in CT but not 6 minute walk due to better age/sex match for 6 minute walk testing and limitations in CT quality. Non-parametric Mann-Whitney tests were performed for age, BSA, and BMI. Chi square tests were performed for variables including race/ethnicity and sex.

Variables		p-value	Least square means	Standard error	95%CI-L	95%CL-U
Age	Old	0.2865	85.1751	1.5736	81.8393	88.5109
	Young		82.6501	1.646	79.1608	86.1393
Genotype	Wildtype	6.18E-06	76.3632	1.5736	73.0274	79.6991
	Heterozygotes		91.462	1.646	87.9727	94.9512

Supplemental Table S2. Linear mixed effects model results for vertical intercepts. Airspaces are larger in *Eln*$^{+/-}$ mice compared to WT across vertical intercepts. There is no difference in airspace size with age. Abbreviations: 95% CI-L:95% confidence interval lower limit, 95%%CI-U: confidence interval upper limit.

References:

See main text for references.
