# Supplementary material for: Airflow Obstruction in Adults with Williams Syndrome and Mice with Elastin Insufficiency
Source: Diagnostics (Basel). 2022 Jun 10;12(6):1438. doi: 10.3390/diagnostics12061438 (PMC9221558; doi:10.3390/diagnostics12061438)
Supplement: Supplementary file 1 [file diagnostics-12-01438-s001.zip › Supplemental Figure S1.pdf]

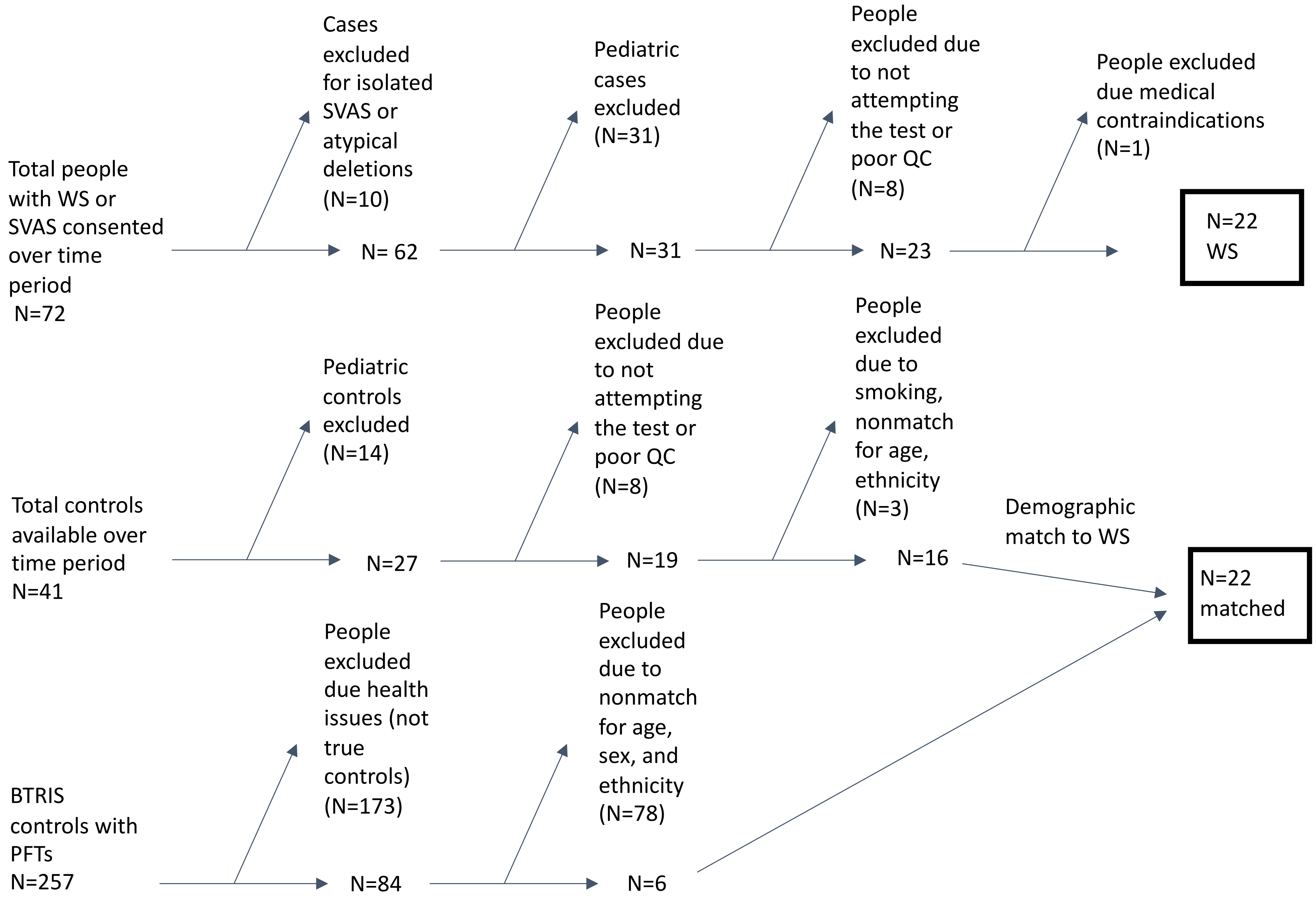

Figure S1. Inclusion and exclusion criteria for the study. Pediatric cases and controls were excluded, as well as those unable to complete the test or those with poor QC. Controls were matched to cases in aggregate by age, sex, and race/ethnicity.
